# Physical activity and short video addiction among college students: the serial mediating roles of self-efficacy and loneliness

**DOI:** 10.3389/fpsyg.2026.1826559

**Published:** 2026-04-27

**Authors:** Enzheng Deng

**Affiliations:** Xi’an Polytechnic University, Xi’an, China

**Keywords:** loneliness, physical activity, self-efficacy, serial mediation, short video addiction

## Abstract

**Objective:**

This study examined the relationship between physical activity and short video addiction among college students, with a particular focus on the mediating roles of self-efficacy and loneliness.

**Methods:**

A cross-sectional survey was conducted among 581 students from three universities. Measures included validated scales assessing physical activity, self-efficacy, loneliness, and short video addiction, with demographic covariates (age, grade, major type [STEM vs. non-STEM], and urban–rural background). Data were analyzed using SPSS 27.0 and Hayes’ PROCESS macro (Model 6). Bootstrap analysis (5,000 resamples) was employed to test mediation effects with 95% confidence intervals.

**Results:**

Physical activity was positively associated with self-efficacy and negatively associated with loneliness and short video addiction. Self-efficacy was negatively associated with both loneliness and short video addiction, whereas loneliness showed a positive association with short video addiction. Mediation analyses showed that physical activity reduced short video addiction both directly (*β* = −0.074, *p* < 0.001) and indirectly through self-efficacy and loneliness, including a serial mediation pathway. The total indirect effect was −0.0330 (95% *CI* [−0.0502, −0.0179]), accounting for 30.8% of the total effect.

**Conclusion:**

Physical activity reduces short video addiction directly and indirectly by enhancing self-efficacy and reducing loneliness, with a significant sequential mediation pathway. These findings highlight potential psychological mechanisms and provide practical implications for interventions targeting short video addiction among college students.

## 1 Introduction

The rapid advancement of mobile internet technologies and algorithm-driven recommendation systems has facilitated the emergence of short videos as a globally influential form of digital entertainment. These videos are characterized by fragmented content, immersive experiences, and immediate feedback features that align closely with the fast-paced information consumption habits of contemporary society (Wang, 2020). By June 2025, China had approximately 1.068 billion short-video users, accounting for 95.1% of all internet users. College students and adolescents constitute the most active demographic groups (Center, C. I. N. I, 2025). Algorithmic recommendation mechanisms, dense informational content, and immediate reward structures are known to activate the brain’s reward circuitry, particularly dopaminergic pathways. This activation fosters immersive engagement, impairs impulse control, and weakens self-regulatory capacities, thereby contributing to excessive and potentially addictive short video use (SVA; Griffiths, 2005; Brand et al., 2014; Smith and Short, 2022). Empirical evidence links SVA to a range of negative outcomes, including sleep disturbances, diminished academic performance, attention deficits, increased depression and anxiety, and reduced subjective well-being (Andreassen, 2015; Kuss and Griffiths, 2017; Ye et al., 2022). Although existing literature has predominantly focused on personality traits, emotional distress, and social environmental risk factors as antecedents of SVA (Qin et al., 2022; Guo and Chai, 2024), comparatively little research has examined lifestyle behaviors, particularly physical activity, as potential protective factors against SVA. Moreover, existing studies have largely examined isolated mediators, while the underlying psychological mechanisms linking physical activity to SVA remain insufficiently understood. Therefore, examining the serial mediating roles of self-efficacy and loneliness may provide deeper insight into the psychological pathways linking physical activity with SVA. The present study addresses this gap by examining how physical activity influences SVA among college students, with a particular focus on the sequential mediating roles of self-efficacy and loneliness. By elucidating these underlying psychological mechanisms, the study aims to provide both theoretical insights and practical guidance for the development of evidence-based interventions targeting SVA.

### 1.1 Physical activity and short video addiction

In the present study, physical activity is conceptualized as the overall level of engagement in bodily movement, regardless of type or context. This operationalization is consistent with prior research emphasizing the cumulative effects of total physical activity on psychological and behavioral outcomes. Engagement in physical activity not only promotes physical health but also enhances psychological well-being, cognitive function, and social adaptation, while strengthening self-regulation and self-control, thereby reducing the risk of problematic digital media use (Busch and McCarthy, 2021; García-Ortiz et al., 2025). Team-based sports (e.g., basketball, football) foster a sense of belonging through cooperation, skill-based activities (e.g., dance, climbing) enhance competence through challenge completion; and self-selected activities promote autonomy in decision-making. From a physiological perspective, regular exercise improves autonomic nervous system function, increases brain-derived neurotrophic factor (BDNF) and glucocorticoid levels, enhances prefrontal cortex gray matter density; and stimulates endogenous endorphin release, collectively reducing reliance on immediate rewards from short videos (Roig et al., 2013). Empirical evidence indicates a significant negative association between physical activity and problematic internet and smartphone use (Park, 2014; Huang et al., 2022). Building on this evidence, systematic reviews suggest that regular physical activity reduces SVA symptoms and improves self-regulatory capacities (Busch and McCarthy, 2021; García-Ortiz et al., 2025). Meta-analytic findings provide more robust support, demonstrating that physical activity interventions significantly alleviate internet addiction symptoms among college students (Yu et al., 2025).

*H*1: Physical activity negatively predicts short video addiction.

### 1.2 The mediating effect of self-efficacy

Self-efficacy refers to an individual’s belief in their capability to successfully accomplish tasks and cope with environmental challenges (Bandura, 1977) and constitutes a core psychological mechanism influencing the initiation, maintenance, and self-regulation of health behaviors (Bandura, 2004). Previous research has consistently shown a positive association between self-efficacy and physical activity, with individuals possessing higher self-efficacy being more likely to adhere to exercise routines (McAuley and Blissmer, 2000). In the context of addictive behaviors, self-efficacy has been shown to predict SVA, including smartphone use and SVA (Du and Zhang, 2022). Individuals with lower self-efficacy are more susceptible to impulsive digital media use, which may further undermine self-regulatory capacities (Lin et al., 2022). From a neurobiological perspective, engaging in physical activity enhances individuals’ confidence in their physical abilities and behavioral control, thereby increasing self-efficacy. Elevated self-efficacy, in turn, helps suppress impulsive media use and reduces the risk of SVA (Fang et al., 2025).

*H*2: Self-efficacy mediates the relationship between physical activity and short video addiction.

### 1.3 The mediating role of loneliness

College students are in a critical stage of social role transition and peer relationship reconstruction, with heightened needs for social belonging and emotional support, making them more susceptible to loneliness. Loneliness is not only a key risk factor for mental health problems but also closely linked to various maladaptive behaviors. According to compensatory internet use theory, individuals may turn to online media to compensate when real-life social connections are lacking or emotional needs are unmet, thereby increasing the risk of addictive behaviors (Davis, 2001). Empirical studies have shown that higher levels of loneliness are associated with smartphone addiction, SVA, and excessive short video consumption (Satici, 2019). Physical activity is an important psychological and social resource for alleviating loneliness. Exercise improves mood and fosters social interaction, cooperation, and a sense of belonging, thereby reducing loneliness. Research indicates a significant negative association between physical activity and loneliness, with regularly active college students reporting lower levels of loneliness (Barker, 2009; Liang et al., 2025). Exercise interventions can also enhance overall mental health by mitigating loneliness.

*H*3: Loneliness mediates the relationship between physical activity and short video addiction.

### 1.4 Serial mediation effects of self-efficacy and loneliness

This hypothesized sequence is grounded in social cognitive theory (Bandura, 1977) and compensatory internet use theory (Davis, 2001). Physical activity, a behavioral resource, enhances personal psychological resources (e.g., self-efficacy), which reduces negative emotional states (e.g., loneliness) that drive compensatory online behaviors. Specifically, behavioral resources, such as physical activity, can enhance personal psychological resources (e.g., self-efficacy), subsequently reducing negative emotional states (e.g., loneliness) that drive compensatory online behaviors. Physical activity provides mastery experiences and enhances perceived competence, thereby strengthening self-efficacy (Dishman et al., 1985). According to social cognitive theory, higher self-efficacy promotes proactive coping, social engagement, and adaptive behavioral regulation. In turn, increased social participation and improved coping capacity reduce feelings of loneliness. From the perspective of compensatory internet use theory, loneliness serves as a key driver of excessive online engagement, as individuals attempt to compensate for unmet social and emotional needs in offline contexts. Therefore, reduced loneliness is expected to decrease reliance on short video platforms, forming a sequential pathway from physical activity to SVA reduction. Regular physical activity enhances perceived competence, achievement experiences, and self-control confidence, thereby boosting self-efficacy. Elevated self-efficacy promotes proactive social engagement, reduces social avoidance, and alleviates loneliness. Empirical evidence shows that physical activity improves mental health via self-efficacy and reduces SVA and addiction through decreased loneliness (Zhu et al., 2025).

*H*4: Self-efficacy and loneliness sequentially mediate the relationship between physical activity and short video addiction.

### 1.5 The present study

This study examines the impact of physical activity on college students’ short video addiction (SVA) and explores the underlying psychological mechanisms through both independent and sequential mediation processes. Specifically, the study addresses three core questions:

- Does physical activity significantly reduce the risk of SVA among college students?
- Do self-efficacy and loneliness independently mediate the relationship between physical activity and SVA?
- Do self-efficacy and loneliness jointly constitute a serial mediation pathway linking physical activity to SVA?

**Figure 1:**
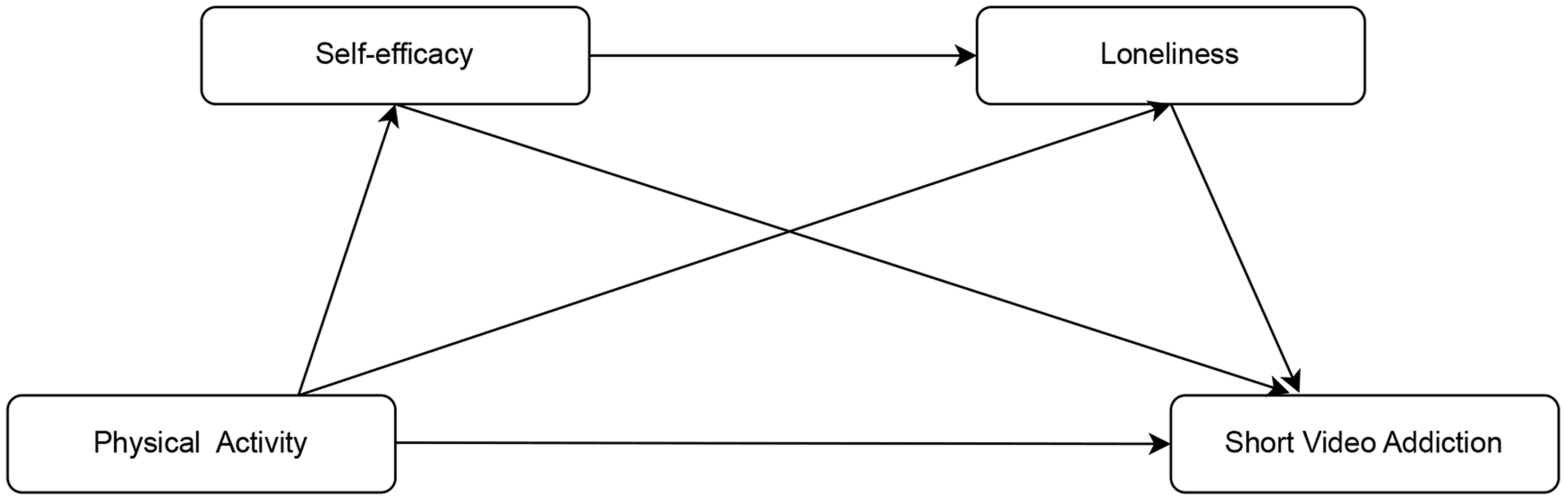
Illustrates the hypothesized relationships among physical activity, self-efficacy, loneliness, and SVA. Physical ctivity (PA) is hypothesized to affect SVA directly and indirectly via elf-fficacy (SE) and oneliness (LON). Arrows indicate the direction of hypothesized effects. PA → SE represents the positive effect of physical activity on self-efficacy; SE → SVA represents the negative effect of self-efficacy on SVA; PA → LON and LON → SVA illustrate the mediation pathway through loneliness.

Grounded in social cognitive theory and compensatory internet use theory, we propose a theoretical model linking physical activity to SVA. Physical activity, as a behavioral resource, enhances personal psychological resources (i.e., self-efficacy). Elevated self-efficacy subsequently alleviates negative emotional states (i.e., loneliness), ultimately reducing short video addiction. This framework offers a more integrative perspective than prior studies that focused on single mediators (see Figure 1).

## 2 Materials and methods

### 2.1 Participants

We conducted this study during the 2026 winter break (January to February). A total of 617 undergraduate and graduate students from three universities in western China were recruited using convenience sampling. Data were collected via the online survey platform Wenjuanxing. QR codes were distributed by faculty members to facilitate participant recruitment. Participants electronically provided informed consent and could withdraw at any time, ensuring that participation was voluntary, anonymous, and strictly confidential. To ensure data quality, responses that were completed implausibly quickly, exhibited patterned responses, or were otherwise invalid were excluded, resulting in a final valid sample of 581 participants (94.2%). The sample exhibited considerable diversity in demographic and academic characteristics. Regarding academic standing, the sample included 120 first-year students (20.7%), 62 sophomores (10.7%), 186 juniors (32.0%), 154 seniors (26.5%), and 59 graduate students (10.2%). By discipline, 457 participants (78.7%) were enrolled in STEM fields, while 124 (21.3%) pursued non-STEM majors. The sample was nearly balanced by residential background: 286 students (49.2%) from urban areas and 295 (50.8%) from rural areas. Age distribution was as follows: 55 participants (9.5%) were 18 years or younger, 213 (36.7%) were 19–20 years old, 225 (38.7%) were 21–22 years old, and 88 (15.1%) were 23 years or older. This heterogeneous sample provides a robust basis for examining the interrelationships between physical activity, self-efficacy, loneliness, and short video addiction, while accounting for variability across demographic and academic variables.

### 2.2 Measures

#### 2.2.1 Physical activity scale

Physical activity was measured using the Physical Activity Rating Scale-3 (PARS-3; Liang, 1994), which assesses exercise intensity, duration, and frequency using 5-point Likert scales. Total physical activity was computed using the following formula: *Exercise = Intensity×(Time − 1) × Frequency*, yielding a composite score that reflects overall activity level, with higher scores indicating greater physical activity. The PARS-3 has been widely used in Chinese populations. In the present study, internal consistency was acceptable (Cronbach’s *α* = 0.714), indicating adequate reliability for assessing participants’ physical activity levels.

#### 2.2.2 Self-efficacy scale

Participants’ self-efficacy was measured using the Chinese version of the General Self-Efficacy Scale (GSES; Wang et al., 2001). The scale consists of 10 items assessing generalized confidence in coping with challenges, rated on a 4-point Likert scale (1 = “not at all true” to 4 = “completely true”), and includes no reverse-coded items. Total scores were calculated by summing item responses, with higher scores indicating greater levels of self-efficacy. In this present study, the GSES demonstrated excellent internal consistency (Cronbach’s *α* = 0.921), indicating its high reliability for assessing self-efficacy in the present sample.

#### 2.2.3 Loneliness scale

In this present study, participants’ loneliness was assessed using the UCLA Loneliness Scale-8 (ULS-8), adapted by Hays and DiMatteo (1987) from the original UCLA-20 scale. The scale consists of eight items, including six positively worded “loneliness” items and two negatively keyed “non-loneliness” items, with the latter being reverse-scored. Items are rated on a 4-point Likert scale (1 = never, 4 = always), yielding a total score ranging from 8–32, with higher scores indicating greater loneliness. The Chinese version employed in this study was translated and validated by bilingual researchers. The scale demonstrated good internal consistency (Cronbach’s *α* = 0.808).

#### 2.2.4 Short video addiction scale

Short video addiction was measured using the Short Video Addiction Scale for College Students (Qin et al., 2022). Although labeled “addiction,” this instrument is intended to assess problematic and excessive patterns of short video use at the behavioral level rather than clinically diagnosed addiction. The 14-item scale assesses four dimensions: loss of control, avoidance, withdrawal, and inefficacy. Items are rated on a 5-point Likert scale (1 = “strongly disagree” to 5 = “strongly agree”), with higher total scores indicating greater addiction tendency. In the present study, the scale demonstrated good internal consistency (Cronbach’s *α* = 0.902), indicating satisfactory reliability for assessing short video addiction among college students.

### 2.3 Statistical analyses

All statistical analyses were performed using SPSS 27.0. Descriptive statistics were calculated to summarize sample characteristics and key study variables. Pearson correlation analyses were conducted to examine bivariate associations among physical activity, self-efficacy, loneliness, and short video addiction, self-efficacy, loneliness, and SVA. Mediation effects were tested using Hayes’ PROCESS macro (Model 6), with bias-corrected bootstrap procedures with 5,000 bootstrap samples to estimate indirect effects and corresponding 95% confidence intervals. To examine potential influence of common method bias, Harman’s single-factor test was conducted. Results indicated that the first unrotated factor accounted for 25.30% of the total variance, below the commonly recommended threshold of 40%, suggesting that common method bias was unlikely to substantially influence the findings. Furthermore, no single factor accounted for the majority of the covariance among the measured variables.

## 3 Results

### 3.1 Descriptive statistics and correlations

Descriptive statistics and bivariate correlations among the main study variables are presented in Table 1. The sample (*N* = 581) reported a mean physical activity score of 26.41 (*SD* = 20.45), a mean self-efficacy score of 25.30 (*SD* = 5.45), a mean loneliness score of 16.16 (*SD* = 4.06), and reported 35.50 (*SD* = 9.52) for short video addiction (SVA). Correlation analyses indicated that physical activity was significantly positively associated with self-efficacy (*r* = 0.145, *p* < 0.01), indicating that students who engaged more frequently and intensively in physical activity tended to report higher levels of perceived competence. Physical activity was significantly negatively correlated with loneliness (*r* = −0.157, *p* < 0.01) and SVA (*r* = −0.239, *p* < 0.01), indicating that higher physical activity was associated with lower loneliness and reduced short video addiction tendencies. Self-efficacy showed a moderate negative correlation with loneliness (*r* = −0.319, *p* < 0.01) and SVA (*r* = −0.276, *p* < 0.01), indicating that individuals with higher self-efficacy were less likely to experience loneliness and short video addiction. In contrast, loneliness was moderately positively correlated with SVA (*r* = 0.404, *p* < 0.01), representing the strongest association among the examined variables. The observed correlation pattern aligns with the hypothesized mediation framework, providing preliminary support for subsequent regression and mediation analyses.

**Table 1:** Correlation analysis between the variables.

Variable	*M*	*SD*	1	2	3	4
Physical activity	26.415	20.450	1	-	-	-
Self-efficacy	25.298	5.450	0.145**	1	-	-
Loneliness	16.160	4.057	−0.157**	−0.319**	1	-
SVA	35.503	9.524	−0.239**	−0.276**	0.404**	1

*N* = 581. **p < 0.01.

### 3.2 Regression analysis and assumption testing for mediation model

Data were analyzed using SPSS 27.0 and Hayes’ (2013) PROCESS macro (Model 6), controlling for age, grade, academic discipline (STEM vs. non-STEM), and urban/rural background as covariates to account for potential confounding effects.

As shown in Table 2, physical activity was significantly negatively associated with SVA *(β* = −0.107, *p* < 0.001), indicating that higher physical activity corresponded to lower short video addiction levels. Concurrently, physical activity (PA) positively predicted self-efficacy (*β* = 0.037, *p* < 0.001) and negatively predicted loneliness (*β =* −0.023, *p* < 0.01), suggesting that physical activity not only enhances individuals’ confidence in managing challenges but also alleviates feelings of social isolation. In line with theoretical expectations, self-efficacy negatively predicted both loneliness (*β =* −0.225, *p* < 0.001) and SVA (*β =* −0.236, *p* < 0.001), whereas loneliness positively predicted SVA (*β* = 0.779, *p* < 0.001), supporting the view of loneliness as a risk factor for addictive digital behaviors. Importantly, after accounting for both mediators simultaneously, PA remained a significant predictor of SVA (*β* = −0.074, *p* < 0.001), indicating partial mediation and suggesting that additional unmeasured mechanisms may also contribute to the protective effect of physical activity.

**Table 2:** Test of chain-mediated model effect.

Effect path	Effect	95% *CI* lower	95% *CI* upper	Effect size (%)
Total effect	−0.1070	−0.1440	−0.0700	100%
Direct effect	−0.0740	−0.1085	−0.0394	69.20%
Total indirect effect	−0.0330	−0.0502	−0.0179	30.80%
Physical activity → Self-efficacy → SVA	−0.0086	−0.0186	−0.0016	8.00%
Physical activity → Loneliness → SVA	−0.0180	−0.0308	−0.0069	16.80%
Physical activity → Self-efficacy → Loneliness → SVA	−0.0064	−0.0117	−0.0020	6.00%

**Figure 2:**
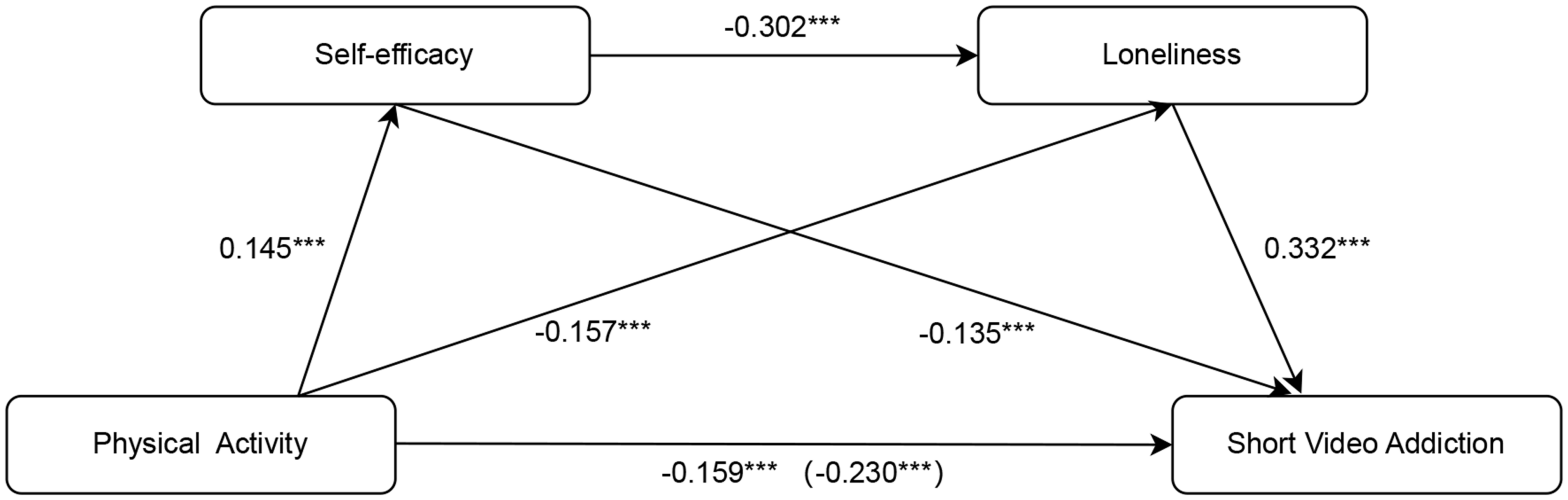
Pathway model of physical activity affecting SVA. Standardized path coefficients are shown along each arrow. Positive and negative signs indicate the direction of effects. Physical activity (PA) is proposed to influence SVA both directly and indirectly through self-efficacy (SE) and loneliness (LON). PA → SE: 0.145***, indicating a positive association between physical activity and self-efficacy. PA → LON: −0.157***, indicating a negative association between physical activity and loneliness. SE → LON: −0.302***, indicating that higher self-efficacy is associated with lower loneliness. SE → SVA: −0.135***, indicating a direct negative effect of self-efficacy on SVA. LON → SVA: 0.332***, indicating a positive association between loneliness and SVA. PA → SVA: −0.230*** (total effect) and −0.159*** (direct effect, controlling for mediators). ***p < 0.001; all coefficients are standardized. PA, Physical activity; SE, Self-efficacy; LON, Loneliness; SVA, Short video addiction.

Bootstrap analyses (5,000 resamples) indicated a significant total indirect effect of physical activity on SVA via self-efficacy and loneliness (Table 2; Figure 2), confirming the mediation. The indirect effect accounted for a substantial portion of the total effect, suggesting that psychological mechanisms play a key role in this relationship.

Further examination of the mediation pathways showed that both self-efficacy and loneliness independently mediated the association between physical activity and SVA. In addition, a significant serial mediation pathway (physical activity → self-efficacy → loneliness → SVA) was observed, indicating that increased self-efficacy may reduce loneliness, which in turn decreases short video addiction.

Among the indirect pathways, the pathway via the mediating effect of loneliness appeared to contribute more strongly than the pathway via self-efficacy, highlighting loneliness as a key mechanism linking physical activity to reduced SVA. All indirect effects were statistically significant, as their bootstrap confidence intervals did not include zero (see Table 3).

**Table 3:** Regression analysis of variables.

Regression equation		Overall fit index			Significance of regression coefficient	
Result variable	Predictive variable	*R*	*R^2^*	*F*	*β*	*t*
SVA	Physical Activity	0.266	0.071	8.785	−0.107	−5.677***
	Age				−0.060	−1.504
	Grade				0.008	0.192
	STEM vs. Non-STEM				0.073	1.764
	Urban–Rural				0.066	1.632
Self-efficacy	Physical Activity	0.208	0.043	5.219	0.037	3.335***
	Age				0.062	1.360
	Grade				−0.003	−0.097
	STEM vs. Non-STEM				−0.053	−1.278
	Urban–Rural				−0.130	−3.157**
Loneliness	Physical Activity	0.344	0.119	12.873	−0.023	−2.927**
	Self-Efficacy				−0.225	−7.542***
	Age				−0.075	−0.989
	Grade				0.039	0.390
	STEM vs. Non-STEM				−0.027	−0.658
	Urban–Rural				−0.016	−0.490
SVA	Physical Activity	0.470	0.221	23.213	−0.074	−4.207***
	Self-Efficacy				−0.236	−3.417***
	Loneliness				0.779	8.446***
	Age				−0.035	−0.935
	Grade				0.002	0.047
	STEM vs. Non-STEM				0.076	1.820
	Urban–Rural				0.043	1.121

*p < 0.05, **p < 0.01, ***p < 0.001.

Beyond the mediation effects, the inclusion of control variables revealed nuanced patterns. Age and grade showed minimal predictive value for SVA, whereas urban/rural background and STEM vs. non-STEM discipline exhibited marginal associations (*βs* = 0.043–0.076, *p* < 0.10), suggesting potential sociocultural influences on digital addiction that warrant further study. Notably, self-efficacy and loneliness consistently emerged as significant mediators regardless of these covariates, highlighting the psychological processes through which physical activity exerts its protective effects.

These findings provide empirical support for a serial mediation model in which physical activity reduces short video addiction both directly and indirectly via self-efficacy and loneliness. The results emphasize that interventions aiming to mitigate short video addiction should promote regular physical activity and incorporate strategies to enhance self-efficacy and reduce feelings of loneliness, potentially yielding a synergistic protective effect. Furthermore, the differential contribution of the indirect paths—particularly the stronger physical activity → Loneliness → short video addiction pathway—offers new insight into the psychological mechanisms underlying the relationship between lifestyle behaviors and digital addiction.

## 4 Discussion

### 4.1 Physical activity and short video addiction

The present findings revealed a robust negative association between college students’ engagement in physical activity and short video addiction, providing empirical support for Hypothesis 1 and confirming previous research (Jianfeng et al., 2024). This pattern aligns with a growing body of literature highlighting the protective role of regular physical exercise against problematic digital media use. Meta-analytic evidence further suggests that structured exercise, whether implemented as a standalone intervention or integrated into broader behavioral programs, can significantly reduce internet addiction among adolescents and young adults, positioning physical activity as an effective and scalable preventive strategy (Zhou et al., 2024). Mechanistically, physical activity functions as a constructive behavioral alternative, redirecting attention away from immediate gratification sources such as short videos and reducing habitual engagement with highly stimulating digital content. From a behavioral economics perspective, short videos provide immediate rewards with minimal effort, whereas physical activity provides delayed but more sustainable psychological rewards. Moderate-to-high intensity exercise, in particular, has consistently been shown to inversely correlate with smartphone and social media addiction, even after controlling for potential confounders such as anxiety, stress, and baseline screen time (Ding et al., 2025). Beyond behavioral substitution, regular physical activity enhances cognitive and emotional self-regulation by strengthening executive functions—including inhibitory control, working memory, and attentional flexibility—and fostering emotional regulation capacities such as frustration tolerance and stress resilience (Suchá et al., 2024). These self-regulatory improvements enable students to resist immediate digital rewards, thereby systematically lowering the risk of short video addiction. Moreover, exercise promotes broader aspects of psychological well-being, including elevated mood, self-efficacy, and social connectedness, which in turn indirectly discourages compensatory short video use for emotional or social needs. These findings suggest that physical activity serves both as a behavioral alternative and as a psychological protective factor against short video addiction. An alternative explanation for the negative association between physical activity and short video addiction is the time displacement effect. Given that time is finite, increased physical activity may directly reduce the time available for short video consumption. While behavioral substitution may partially account for the observed relationship, it is unlikely to fully explain the findings. Specifically, the significant mediating roles of self-efficacy and loneliness suggest that intrinsic psychological mechanisms are critical. Physical activity not only occupies time but also enhances perceived competence and reduces negative emotional states, thereby decreasing reliance on short video platforms. Therefore, the relationship between physical activity and short video addiction is better understood as a combination of behavioral time allocation and psychological processes, rather than a simple substitution effect.

### 4.2 Mediating role of self-efficacy

The present study provides robust evidence that self-efficacy significantly mediates the relationship between physical activity and short video addiction, providing empirical support for Hypothesis 2. Specifically, regular physical activity appears to enhance individuals’ self-efficacy, which, in turn, is negatively associated with short video addiction (Caplan, 2007; Du and Zhang, 2022). This finding underscores self-efficacy as a central psychological mechanism through which exercise protects against maladaptive digital behaviors. The mediating role of self-efficacy aligns with prior research indicating that adolescents’ exercise intentions influence digital addiction through perceived competence, suggesting that the cognitive and motivational benefits of physical activity extend beyond immediate behavioral engagement. Meta-analytic evidence further corroborates this relationship, showing a consistent positive association between physical activity and self-efficacy, indicating that sustained participation in exercise not only strengthens individuals’ physical health but also enhances their perceived capability to manage challenges and regulate behavior effectively (Xie et al., 2025). Structural equation modeling studies similarly demonstrate that regulatory emotional self-efficacy partially mediates the link between social anxiety and smartphone addiction, indicating that individuals with higher self-efficacy can better resist addictive impulses and navigate digital temptations in socially or emotionally stressful contexts (Xiao and Huang, 2022). Moreover, serial mediation analyses suggest a reciprocal dynamic: students exhibiting higher levels of short video addiction report lower self-efficacy, which may subsequently reduce their engagement in physical activity, creating a reinforcing feedback loop that diminishes protective factors and exacerbates vulnerability to digital overuse (Zhao and Kou, 2024). Beyond these direct effects, enhancing self-efficacy through physical activity likely exerts broader cognitive and emotional benefits, including improved self-regulation, goal-setting, frustration tolerance, and stress resilience, all of which may reduce reliance on short videos as a compensatory coping mechanism. These findings highlight self-efficacy as a key psychological mechanism through which physical activity protects against short video addiction.

### 4.3 Mediating role of loneliness

The present study found that loneliness significantly mediates the relationship between physical activity and short video addiction, consistent with prior research. Empirical evidence indicates that physical activity is negatively associated with loneliness (Xu and Tang, 2024), whereas loneliness shows a strong positive association with smartphone and broader internet addiction, including excessive engagement with short video platforms (Fukuya et al., 2025). This establishes a coherent psychological pathway in which regular physical activity reduces loneliness, which in turn decreases compensatory reliance on highly engaging digital content. Among the three indirect pathways identified in this study, the PA → loneliness → short video addiction pathway accounted for the largest proportion of the indirect effect (16.8%), highlighting loneliness as the key psychological mediator linking physical activity to short video addiction. This finding suggests that reducing loneliness is a key mechanism through which physical activity protects college students from developing short video addiction. Mechanistically, physical activity alleviates loneliness by promoting real-life social interactions, enhancing a sense of belonging, and fostering supportive peer networks. Additionally, exercise improves self-efficacy, self-esteem, and emotion regulation capacities, collectively enhancing individuals’ ability to manage negative affect and stress without relying on digital substitutes for social or emotional fulfillment. Drawing on compensatory internet use theory and cognitive-behavioral models, individuals experiencing loneliness are more likely to seek immediate emotional relief and social validation through short video platforms, reinforcing habitual use and increasing addiction risk (Öztekin et al., 2025). This study positions loneliness as a critical psychological mechanism through which physical activity influences short video addiction, extending prior research focused on internet or smartphone overuse. Findings indicate that physical activity reduces addiction by reducing loneliness, thereby decreasing the need for compensatory online interaction, consistent with previous evidence that exercise indirectly reduces problematic internet use through loneliness reduction (Xu and Tang, 2024). Notably, loneliness may also indirectly exacerbate addictive tendencies through self-depletion and reduced self-control, forming a “loneliness-reduced real-world engagement-and increased online dependence” vicious cycle. These findings underscore loneliness as a central socio-emotional mechanism linking physical activity to reduced short video addiction.

### 4.4 Serial mediation of self-efficacy and loneliness

The present study shows that physical activity not only directly reduces the risk of SVA but also has indirect effects via general self-efficacy and loneliness. Consistent with prior research linking loneliness to behavioral addiction and psychological distress (Erzen and Çikrikci, 2018; Luchetti et al., 2020), our findings suggest that regular participation in physical activity enhances individuals’ general self-efficacy (Peng et al., 2025), which in turn improves coping with academic, social, and daily life challenges. Increased self-efficacy facilitates effective coping and a sense of personal agency, reducing feelings of loneliness. Reduced loneliness then diminishes excessive reliance on online social platforms, establishing a sequential mediation pathway: increased self-efficacy → decreased loneliness → lower SVA risk. The serial mediation pathway aligns with compensatory internet use theory and emotion-cognition regulation frameworks. These frameworks emphasize that individuals may seek online engagement to compensate for unmet social or emotional needs, and that self-regulatory capacity is central for managing affective responses (Wongpakaran et al., 2021). Furthermore, the results support prior findings that self-efficacy functions as a psychological resource, buffering the adverse effects of loneliness while enhancing real-world social experiences (Lee et al., 2023). These findings provide strong support for a serial mediation model in which self-efficacy and loneliness jointly explain how physical activity reduces SVA. Notably, the cross-sectional design of the present study limits causal inference, underscoring the importance of longitudinal or experimental investigations to validate the dynamic process of this serial mediation and to elucidate the temporal interplay between physical activity, self-efficacy, loneliness, and addictive behaviors. Such evidence could inform the design of comprehensive, evidence-based interventions that target psychological, behavioral, and social determinants of SVA among college students.

### 4.5 Theoretical contributions

This study makes three key contributions to the literature. First, while prior studies have primarily examined single mediators (e.g., self-control, stress, or social anxiety), this study integrates self-efficacy and loneliness within a serial mediation framework, providing a more complete understanding of how behavioral resources contribute to reduced digital addiction. Second, the findings identify loneliness as the strongest mediating pathway, underscoring the importance of social–emotional mechanisms in short video addiction. Third, the study adds to research on lifestyle-based prevention strategies by demonstrating that physical activity may function as both a behavioral substitute and a psychological resource in mitigating digital addiction.

### 4.6 Research implications

(a) Promoting college students’ mental health.

These findings have important real-world implications for college students, who are in a critical developmental stage marked by increasing academic demands, identity exploration, and restructuring of social relationships. These transitions often make students especially susceptible to declines in self-efficacy and increases in loneliness, both of which are empirically linked to maladaptive coping behaviors such as short video addiction. Physical activity serves as a practical, accessible, and scalable intervention within university settings. Unlike purely psychological interventions, it simultaneously targets behavioral engagement, cognitive resources, and emotional well-being. By enhancing self-efficacy through mastery experiences and reducing loneliness via social interaction and group participation, physical activity can address both the cognitive and emotional contributors to short video addiction. Therefore, promoting regular physical activity among college students may serve as an effective, low-cost strategy to improve mental health and reduce digital addiction in real-world contexts.

(b) Informing intervention strategies.

These findings support the development of multidimensional, integrated intervention programs in universities. Physical activity serves as a compensatory health behavior to reduce excessive digital media use, including short video platforms (Xu and Tang, 2024); enhancing self-efficacy strengthens psychological resources, lowering addiction risk (Jia et al., 2024); interventions targeting social support and sense of belonging further alleviate loneliness and reduce addiction (Liu et al., 2025). Integrating physical exercise, self-efficacy training, and loneliness interventions into university mental health education and counseling provides a theoretically grounded, evidence-based approach. For example, universities could incorporate structured physical activity programs such as team sports leagues, group fitness classes, or peer-led exercise initiatives, which may simultaneously enhance social connectedness and psychological resources while reducing excessive use of short video platforms. Building on the above findings, interventions should adopt a multidimensional approach that integrates physical activity promotion with strategies to enhance self-efficacy and reduce loneliness. Physical activity not only serves as a behavioral alternative to excessive screen use but also strengthens psychological resources—particularly self-efficacy—that facilitate better self-regulation. In addition, fostering supportive social environments and promoting real-world interpersonal interactions may effectively alleviate loneliness, thereby reducing compensatory use of short video platforms for social and emotional needs. Taken together, interventions that simultaneously target behavioral engagement, psychological resources, and social connectedness may produce synergistic effects in reducing short video addiction among college students.

(c) Advancing multi-level social support systems.

Reducing short video addiction requires coordinated efforts across universities, families, and society. Real-life social support plays a critical protective role in buffering stress and improving emotional outcomes. It also mitigates digital and behavioral addiction, including short video addiction. Individuals receiving higher social support demonstrate stronger adaptation and lower addiction risk. Universities should enhance counseling and support services. Families and communities should reinforce real-life interactions and provide emotional support. Policymakers should optimize campus sports resources and digital ecosystems to establish structural support environments. These efforts collectively help limit youth overreliance on virtual spaces.

### 4.7 Limitations and future directions

This study provides evidence for the protective role of physical activity against SVA among college students and verifies the sequential mediation of self-efficacy and loneliness. Despite these contributions, several limitations warrant consideration. First, the cross-sectional design, based on single-time self-reported data, limits the ability to draw causal inferences. Moreover, data were collected during the winter vacation, when students’ living environments and daily routines differed from the regular academic term. This may affect both the representativeness and ecological validity of the responses. Second, relying on self-report questionnaires may introduce social desirability and recall biases, which can result in measurement error. In addition, the sample was drawn from three universities in western China, potentially limiting the generalizability of the findings. The study did not differentiate among types, frequency, or intensity of physical activity. It also did not account for potential moderating factors, such as personality traits, motivations for short video use, and psychological stress. These omissions may limit the explanatory power of the results. In addition, the present study did not directly measure time allocation between physical activity and short video use, which makes it difficult to disentangle the relative contributions of time displacement and intrinsic psychological mechanisms. Importantly, SVA in this study was assessed using a self-report behavioral scale and captures problematic and excessive short video use patterns rather than clinically diagnosed addiction. Therefore, the findings should be interpreted with appropriate caution. Future research should adopt longitudinal or experimental designs to examine causal mechanisms. It should also expand sample sizes to include students from diverse regions and educational contexts and integrate objective measures of physical activity and short video use, for example through wearable devices or behavioral tasks. Furthermore, additional mediating or moderating variables, such as emotion regulation, social support, or self-control, should be incorporated to examine the robustness and universality of the serial mediation model. Based on self-efficacy theory, interventions could be developed to enhance physical activity participation, strengthen self-regulatory capacity, and alleviate loneliness, thereby reducing short video addiction tendencies among college students. Such interventions would provide theoretical and practical guidance for developing evidence-based prevention strategies in higher education.

## 5 Conclusion

This study provides empirical evidence supporting the protective role of physical activity in mitigating SVA among college students and further elucidates the sequential mediation of self-efficacy and loneliness. The findings suggest that regular and sustained engagement in physical activity directly reduces the risk of SVA. It also indirectly lowers SVA risk by enhancing self-efficacy and alleviating feelings of loneliness. These results highlight the importance of integrating behavioral and psychological strategies in intervention programs. Students should be encouraged to establish consistent exercise routines, while counseling and peer support can promote self-efficacy and social connectedness. Overall, this study advances understanding of the psychological mechanisms underlying SVA and provides theoretical and practical guidance for developing evidence-based prevention and intervention strategies in higher education.

## Data Availability

The raw data supporting the conclusions of this article will be made available by the authors, without undue reservation.
